# Higher education is associated with a better rheumatoid arthritis outcome concerning for pain and function but not disease activity: results from the EIRA cohort and Swedish rheumatology register

**DOI:** 10.1186/s13075-015-0836-6

**Published:** 2015-11-06

**Authors:** Xia Jiang, Maria E. C. Sandberg, Saedis Saevarsdottir, Lars Klareskog, Lars Alfredsson, Camilla Bengtsson

**Affiliations:** Unit of Cardiovascular disease, Institute of Environmental Medicine, Karolinska Institute, Box 210, Stockholm, 171 77 Sweden; Unit of Rheumatology, Department of Medicine, Karolinska University Hospital, Stockholm, Sweden; Center for Occupational and Environmental Medicine, Stockholm County Council, Stockholm, Sweden

**Keywords:** Rheumatoid arthritis, Educational status, Prognosis, DAS28, VAS-pain, HAQ

## Abstract

**Introduction:**

Whether low socioeconomic status (SES) is associated with worse rheumatoid arthritis (RA) outcomes in countries with general tax-financed healthcare systems (such as Sweden) remains to be elucidated. Our aim was to investigate the influence of educational background (achieving university/college degree (high) or not (low)) on the outcomes of early RA, in terms of disease activity (DAS28), pain (VAS-pain), and functional impairment (HAQ).

**Methods:**

We evaluated DMARD-naïve RA patients recruited in the Epidemiological Investigation of RA (EIRA) study with outcomes followed in the Swedish Rheumatology Quality (SRQ) register (N = 3021). Outcomes were categorized in three ways: 1) scores equal to/above median vs. below median; 2) DAS28-based low disease activity, good response, remission; 3) scores decreased over the median vs. less than median. Associations between educational background and outcomes were calculated by modified Poisson regressions, at diagnosis and at each of the three standard (3, 6, 12 months) follow-up visits.

**Results:**

Patients with different educational background had similar symptom durations (195 days) and anti-rheumatic therapies at baseline, and comparable treatment patterns during follow-up. Patients with a high education level had significantly less pain and less functional disability at baseline and throughout the whole follow-up period (VAS-pain: baseline: 49 (28-67) vs. 53 (33-71), p <0.0001; 1-year visit: RR = 0.81 (95 % CI 0.73-0.90). HAQ: baseline: 0.88 (0.50-1.38) vs. 1.00 (0.63-1.50), p = 0.001; 1-year visit: 0.84 (0.77-0.92)). They also had greater chances to achieve pain remission (VAS-pain ≤20) after one year (1.17 (1.07-1.28)). Adjustments for smoking and BMI altered the results only marginally. Educational background did not influence DAS28-based outcomes.

**Conclusion:**

In Sweden, with tax-financed, generally accessible healthcare system, RA patients with a high education level experienced less pain and less functional disability. Further, these patients achieved pain remission more often during the first year receiving standard care. Importantly, education background affected neither time to referral to rheumatologists, disease activity nor anti-rheumatic treatments.

## Introduction

Low socioeconomic status (SES), measured in a variety of ways (e.g., formal educational background, occupation, household income, ethnicity), has been associated with an increased risk of rheumatoid arthritis (RA), and a worse disease outcome [1, 2]. Despite several reports describing the adverse link between deprivation and prognosis [3–11], a majority of the reported studies had a cross-sectional design lacking subsequent follow up [3–6], whereas the existing longitudinal studies have focused mainly on long-term consequences (>2 years from baseline) [7–11]. In none of the previous studies has there been the opportunity to analyze the contribution of other known determinants such as the use of anti-rheumatic therapy, or smoking, on RA outcomes. Additionally, whether low SES is associated with worse RA outcomes in countries with general tax-financed healthcare systems (such as Sweden) remains to be elucidated.

Our aim was to evaluate whether SES, here measured as formal educational background, affects the chance of good control of RA during the first year in patients receiving standard care in Sweden. We used data from a well-established population-based RA cohort that has included incident cases captured before the initiation of the first disease-modifying anti-rheumatic drug (DMARD).

## Methods

### Study population

The study participants were newly diagnosed, DMARD-naive RA patients from the Epidemiological investigation of rheumatoid arthritis (EIRA) study in Sweden (previously described in detail [12]), who were included during 1996–2011, and who had clinical follow-up data from the Swedish Rheumatology Quality (SRQ) register until 2013. In total, 3,021 (92 %) of all EIRA cases were included. We excluded: EIRA patients not reported to SRQ, or despite being reported to SRQ, lacking information on all outcome variables (n = 254); patients with symptom duration longer than one year (n = 18); and one patient who lacked information on educational background. All participants gave informed consent and the study was approved by the ethical review board at Karolinska Institutet.

### Definition of education and potential confounders

Information on exposure (formal educational background) and several confounders (e.g., age at diagnosis, sex, smoking, alcohol consumption, body mass index (BMI), etc.) was collected through a self-administrated questionnaire (from the EIRA study) at baseline. Briefly, age at diagnosis was a continuous variable. Ever smokers were defined as current and former cigarette smokers while never smokers reported they had never smoked. Pack-years of smoking were calculated with one pack-year equivalent to smoking 20 cigarettes per day for one year. Ever drinking was captured by questions about present alcohol consumption and previous habitual consumption, and included both current and former drinkers. Total alcohol consumption was measured in drinks per week (1 drink = 16 g alcohol). BMI was calculated from self-reported height and weight (kg/m^2^). Formal educational background at diagnosis was categorized as having a university/college degree (high level of education) versus no university/college degree (low level of education). Several potential determinants of outcomes, including time to the first encounter with a rheumatologist, severity of disease at baseline, first and subsequent use of anti-rheumatic therapy, were recorded by rheumatologists and were accessible through SQR.

### Outcome measures

Three clinical aspects were considered as outcome measures: the 28-joint disease activity score (DAS28), visual analog scale for pain (VAS-pain, range 0–100), and health assessment questionnaire (HAQ) score (range 0–3). Please note that in all measures, a higher score represents a poorer outcome.

We primarily classified all outcome measures into equal to/above median versus below median. We further separately categorized patients based on achievement (yes/no) of low disease activity (DAS28 ≤3.2), good response (DAS28 ≤3.2 and DAS28 decrease >1.2) and remission (DAS28 ≤2.6) according to European League Against Rheumatism (EULAR)/American College of Rheumatology (ACR) recommendations [13] and defined pain remission as ≤20 mm on a 100-mm VAS [14]. We further looked into the decrease (scores from each visit compared to the baseline level) larger than the median decrease in all outcome measures. We also investigated the influence of educational background on each component of the DAS28 (i.e., the number of tender and swollen joints out of 28 joints, the erythrocyte sedimentation rate (ESR), and the patient’s global assessment of general health).

### Statistical analysis

We used modified Poisson regression to obtain the risk ratio (RR) with 95 % confidence interval (CI) of the outcomes associated with educational background [15]. Patients with university/college degree (high level of education, exposed) were compared with patients without (low level of education, unexposed), at each time point, i.e., the first three follow-up visits (3 (2.5–5.0), 6 (5.0–7.5), and 12 (7.5–18.0) months) after diagnosis.

All analyses were adjusted for potential confounders, i.e., age at diagnosis (continuous, fitted in both the linear and squared term in the model), sex (binary), pack-years of cigarettes smoking (continuous), total alcohol assumption (continuous), BMI (continuous), and outcome measure at baseline (e.g., when analyzing DAS-28-related outcomes at each of the three follow-up visits, we also adjusted for DAS28 baseline level. The same strategy was applied for the HAQ and VAS-pain). In addition, we performed analysis with additional adjustments for residential area (county), leisure time physical activity, anti-citrullinated protein/peptide antibodies (ACPA)-status, treatment at baseline, and study phase. We subsequently carried out stratified analysis based on ACPA status (ACPA-positive and ACPA-negative subgroups), and treatment (DMARDs and other treatments). All analyses were performed in SAS V.9.3.

## Results

Basic characteristics of the patients are shown in Table 1. In total, 72 % of the cases were female, and 66 % were ACPA-positive. The RA patients with a low level of education were slightly older, comprised more male patients, and were more likely to be smokers, overweight, and have a less sedentary lifestyle as compared with university-educated patients; whereas in terms of ACPA status, no differences were observed between the two groups. In addition, the median symptom duration was 195 days, exactly the same for both groups of patients.

**Table 1:** Baseline characteristics of patients included in the study, N = 3021

	With university/college degree	Without university/college degree	Total	Chi square statistics, *p* values
A: Basic characteristics	number (%)	number (%)	number (%)	
Sex				
Female	586 (82.4)	1585 (68.6)	2171 (71.9)	51.2, <0.0001
Male	125 (17.6)	725 (31.4)	850 (28.1)	
Age (years) at diagnosis				
<40	164 (23.1)	410 (17.8)	574 (19.0)	44.6, <0.0001
40–50	160 (22.5)	370 (16.0)	530 (17.5)	
50–60	215 (30.2)	694 (30.0)	909 (30.1)	
60–70	172 (24.2)	836 (36.2)	1008 (33.4)	
Median	51.0	52.4	54.5	
Treatment initiated at the diagnosis				
DMARDs (including MTX)	606 (85.2)	2004 (86.8)	2610 (86.4)	1.1, 0.30
Biologics	32 (4.5)	145 (6.3)	177 (5.9)	3.1, 0.08
Cortisone	276 (38.8)	946 (41.0)	1223 (40.5)	1.0, 0.32
NSAIDs	384 (54.0)	1153 (49.9)	1537 (50.9)	0.6, 0.06
ACPA status				
Present	458 (65.2)	1521 (66.8)	1979 (66.4)	0.6, 0.44
Absent	245 (34.8)	757 (33.2)	1002 (33.6)	
Cigarette smoking				
Never	297 (41.8)	674 (29.3)	971 (32.4)	76.0, <0.0001
Past smokers	216 (30.5)	701 (30.5)	917 (30.4)	
Current smokers	106 (14.9)	682 (29.7)	788 (26.2)	
Non regular smokers	91 (12.8)	242 (10.5)	333 (11.0)	
Alcohol consumption				
Never drinkers	49 (6.9)	266 (11.5)	315 (10.4)	12.5, 0.0004
Ever drinkers	662 (93.1)	2041 (88.5)	2703 (89.6)	
BMI				
Normal weight: BMI <25	445 (63.9)	1103 (48.6)	1548 (52.2)	51.1, <0.0001
Overweight: BMI 25–30	189 (27.1)	821 (36.2)	1010 (34.1)	
Obese: BMI >30	63 (9.0)	344 (15.2)	407 (13.7)	
Median	23.7	25.1	24.8	
Physical activity before diagnosis				
Sedentary/moderate exercise	518 (73.0)	1513 (65.8)	2031 (67.5)	12.7, 0.0004
Regular exercise/work out	192 (27.0)	787 (34.2)	979 (32.5)	
B: Outcome measures	Median (IQR)	Median (IQR)	Median (IQR)	*P* values (Wilcoxon test)
DAS28 at diagnosis	5.23 (4.26 to 6.05)	5.21 (4.32 to 6.07)	5.22 (4.30 to 6.06)	0.93
VAS-pain at diagnosis	49 (28 to 67)	53 (33 to 71)	51 (32 to 70)	<0.0001
HAQ at diagnosis	0.88 (0.50 to 1.38)	1.00 (0.63 to 1.50)	1.00 (0.63 to 1.38)	0.001
DAS28 change at 3-month visit	–1.69 (–2.80 to –0.72)	–1.63 (–2.72 to –0.62)	–1.65 (–2.74 to –0.63)	0.19
VAS-pain change at 3-month visit	–17 (–40 to –2)	–20 (–43 to –1)	–19 (–42 to –1)	0.69
HAQ change at 3-month visit	–0.38 (–0.75 to 0.00)	–0.37 (–0.75 to 0.00)	–0.37 (–0.75 to 0.00)	0.28
DAS28 change at 6-month visit	–2.19 (–3.13 to –1.01)	–2.02 (–3.06 to –0.91)	–2.05 (–3.08 to –0.93)	0.19
VAS-pain change at 6-month visit	–20 (–41 to –2)	–21 (–43 to –1)	–21 (–42 to –1)	0.94
HAQ change at 6-month visit	–0.50 (–0.88 to –0.12)	–0.38 (–0.87 to 0.00)	–0.38 (–0.87 to 0.00)	0.19
DAS28 change at 1-year visit	–2.37 (–3.40 to –1.28)	–2.16 (–3.30 to –0.96)	–2.21 (–3.32 to –1.03)	0.02
VAS-pain change at 1-year visit	–20 (–42 to –5)	–22 (–43 to 0)	–21 (–43 to –1)	0.72
HAQ change at 1-year visit	–0.50 (–0.88 to –0.13)	–0.38 (–0.88 to 0.00)	–0.38 (–0.88 to 0.00)	0.02

*DMARD* disease-modifying anti-rheumatic drug, *MTX* methotrexate, *NSAID* non-steroidal anti-inflammatory drug, *ACPA* anti-citrullinated protein/peptide antibodies, *BMI* body mass index, *DAS28* disease activity in 28 joints, *VAS* visual analog scale, *HAQ* health assessment questionnaire

DAS28-based outcome measures showed comparable baseline levels between the exposed (patients with a university education) and unexposed (patients without a university education) groups but for VAS-pain and HAQ, slightly, although statistically significantly lower, median levels were observed among patients with a university education (VAS-pain 49 (28–67) vs. 53 (33–71), *p* value <0.0001, HAQ 0.88 (0.50–1.38) vs. 1.00 (0.63–1.50), *p* value = 0.001). We observed no significant differences in anti-rheumatic treatments initiated at baseline or during the first year of disease (Table 2).

**Table 2:** Prescription of anti-rheumatic therapy among patients with different levels of education

Treatment	Without degree, number (%)	With degree, number (%)	Crude *p* values, from Chi square test	Adjuste*d p* values*
Treatment initiated at the diagnosis (baseline)				
DMARDs	2004 (86.8)	606 (85.2)	0.30	0.44
NSAIDs	1153 (49.9)	384 (54.0)	0.06	0.10
Cortisone	946 (41.0)	276 (38.8)	0.32	0.69
Biologics	145 (6.3)	32 (4.5)	0.08	0.08
3-month visit				
DMARDs	1642 (90.7)	518 (89.8)	0.52	0.59
NSAIDs	699 (38.6)	241 (41.8)	0.17	0.10
Cortisone	736 (40.6)	242 (41.9)	0.58	0.47
Biologics	186 (10.3)	52 (9.0)	0.38	0.27
6-month visit				
DMARDs	1435 (90.4)	409 (90.3)	0.93	0.56
NSAIDs	558 (35.2)	165 (36.4)	0.62	0.26
Cortisone	606 (38.2)	173 (38.2)	0.99	0.89
Biologics	203 (12.8)	56 (12.4)	0.81	0.42
1-year visit				
DMARDs	1807 (87.7)	553 (87.5)	0.91	0.33
NSAIDs	660 (32.0)	190 (30.1)	0.35	0.82
Cortisone	711 (34.5)	223 (35.3)	0.72	0.86
Biologics	357 (17.3)	120 (19.0)	0.34	0.65

*P values were adjusted for age at diagnosis, gender, alcohol assumption, pack-years of smoking, body mass index, and baseline outcome values. DMARDs disease-modifying anti-rheumatic drugs, NSAIDs nonsteroidal anti-inflammatory drugs

### Risk ratios for outcome measures equal to/above median

Throughout the follow-up period, patients with a high level of education had a decreased risk of having VAS-pain above the median compared with patients with a low level of education (RR (95 % CI) for the 3-month visit 0.82 (0.74–0.91); 6-month visit 0.86 (0.77–0.96); 1-year visit 0.81 (0.73–0.90)). They also had less functional impairment than patients with a low level of education (HAQ, 3-month visit 0.83 (0.75–0.91); 6-month visit 0.87 (0.78–0.97); 1-year visit 0.84 (0.77–0.92)) (Table 3). For both VAS-pain and HAQ, the results at the 3-month and 1-year visits remained significant after adjustment. No significant associations were identified between educational background and DAS28.

**Table 3:** Risk ratios and 95 % confidence intervals for the association between educational level and having outcome measures of rheumatoid arthritis above the median

Visits	University/college degree	Outcome values above the median			
		No	Yes	Risk ratio, crude (95 % CI)	Risk ratio* (95 % CI)
DAS28					
3 months	No	860 (74.7)	291 (25.3)	1.0 ref.	1.0 ref.
	Yes	889 (76.6)	272 (23.4)	0.95 (0.86 to 1.05)	0.99 (0.90 to 1.09)
6 months	No	762 (77.0)	227 (23.0)	1.0 ref.	1.0 ref.
	Yes	776 (78.4)	214 (21.6)	0.96 (0.86 to 1.07)	1.01 (0.90 to 1.13)
1 year	No	975 (75.6)	314 (24.4)	1.0 ref.	1.0 ref.
	Yes	1007 (77.4)	294 (22.6)	0.95 (0.87 to 1.04)	0.98 (0.89 to 1.08)
VAS-pain					
3 months	No	817 (72.1)	316 (27.9)	1.0 ref.	1.0 ref.
	Yes	948 (79.3)	247 (20.7)	0.82 (0.74 to 0.91)	0.88 (0.79 to 0.98)
6 months	No	751 (75.3)	246 (24.7)	1.0 ref.	1.0 ref.
	Yes	804 (80.3)	197 (19.7)	0.86 (0.77 to 0.96)	0.93 (0.82 to 1.05)
1 year	No	947 (72.9)	352 (27.1)	1.0 ref.	1.0 ref.
	Yes	1058 (80.0)	264 (20.0)	0.81 (0.73 to 0.90)	0.85 (0.76 to 0.94)
HAQ					
3 months	No	743 (71.8)	292 (28.2)	1.0 ref.	1.0 ref.
	Yes	986 (79.1)	261 (20.9)	0.83 (0.75 to 0.91)	0.90 (0.82 to 0.99)
6 months	No	689 (75.1)	229 (24.9)	1.0 ref.	1.0 ref.
	Yes	827 (80.0)	207 (20.0)	0.87 (0.78 to 0.97)	0.93 (0.83 to 1.04)
1 year	No	817 (72.9)	304 (27.1)	1.0 ref.	1.0 ref.
	Yes	1130 (79.5)	292 (20.5)	0.84 (0.77 to 0.92)	0.90 (0.82 to 0.98)

Risk ratio crude: poisson regression estimates without adjustment. Risk ratio*: poisson regression estimates adjusted for age at diagnosis, gender, alcohol assumption, pack-years of smoking, body mass index, and baseline outcome values. *DAS28* disease activity in 29 joints, *VAS* visual analog scale

### Risk ratios for DAS28 remission, VAS-pain remission

We observed no associations between educational background and DAS28 remission, low disease activity or good response, except for good response at the 1-year visit (1.10 (1.01–1.20); this did not remain significant after adjustment for potential confounders) and for remission at the 1-year visit (1.12 (1.02–1.24)). Neither did we observe any significant associations for DAS28 components (ESR, C-reactive protein (CRP), and patient global assessment) in relation to educational background (data not shown). Higher education was associated with 15–25 % greater chance of achieving VAS-pain remission (3-month visit: 1.25 (1.13–1.38); 6-month visit: 1.14 (1.02–1.28); 1-year visit: 1.17 (1.07–1.28)). The results, however, did not remain significant at the 6-month visit after adjustment (Table 4).

**Table 4:** Risk ratios and 95 % confidence intervals for the association between educational level and having outcome measures of rheumatoid arthritis decrease over the median, or achieve remission

Visits	University/college degree	Outcome values decrease over the median or achieve remission			
		No	Yes	Risk ratio crude (95 % CI)	Risk ratio* (95 % CI)
DAS28 low disease activity					
3 months	No	960 (76.4)	297 (23.6)	1.0 ref.	1.0 ref.
	Yes	789 (74.8)	266 (25.2)	1.05 (0.95 to 1.16)	1.00 (0.91 to 1.11)
6 months	No	737 (78.2)	206 (21.8)	1.0 ref.	1.0 ref.
	Yes	801 (77.3)	235 (22.7)	1.02 (0.93 to 1.13)	0.98 (0.88 to 1.09)
1 year	No	822 (78.2)	229 (21.8)	1.0 ref.	1.0 ref.
	Yes	1160 (75.4)	379 (24.6)	1.07 (0.99 to 1.14)	1.04 (0.97 to 1.13)
DAS28 good response					
3 months	No	1073 (75.9)	340 (24.1)	1.0 ref.	1.0 ref.
	Yes	642 (75.3)	211 (24.7)	1.02 (0.91 to 1.16)	0.98 (0.86 to 1.11)
6 months	No	829 (78.1)	233 (21.9)	1.0 ref.	1.0 ref.
	Yes	682 (77.8)	195 (22.2)	1.01 (0.90 to 1.14)	0.96 (0.85 to 1.08)
1 year	No	964 (78.5)	264 (21.5)	1.0 ref.	1.0 ref.
	Yes	960 (75.0)	320 (25.0)	1.10 (1.01 to 1.20)	1.07 (0.98 to 1.17)
DAS28 remission					
3 months	No	1214 (76.6)	371 (23.4)	1.0 ref.	1.0 ref.
	Yes	535 (73.6)	192 (26.4)	1.11 (0.97 to 1.28)	1.11 (0.97 to 1.28)
6 months	No	962 (78.3)	267 (21.7)	1.0 ref.	1.0 ref.
	Yes	576 (76.8)	174 (23.2)	1.05 (0.92 to 1.20)	1.00 (0.87 to 1.15)
1 year	No	1141 (78.2)	318 (21.8)	1.0 ref.	1.0 ref.
	Yes	841 (74.4)	290 (25.6)	1.12 (1.02 to 1.24)	1.11 (1.00 to 1.23)
VAS-pain decrease over the median					
3 months	No	831 (75.3)	273 (24.7)	1.0 ref.	1.0 ref.
	Yes	882 (76.8)	266 (23.2)	0.96 (0.87 to 1.06)	1.06 (0.97 to 1.16)
6 months	No	750 (77.9)	213 (22.1)	1.0 ref.	1.0 ref.
	Yes	763 (78.3)	212 (21.7)	0.99 (0.89 to 1.10)	1.08 (0.98 to 1.19)
1 year	No	938 (75.9)	298 (24.1)	1.0 ref.	1.0 ref.
	Yes	991 (77.5)	288 (22.5)	0.96 (0.87 to 1.05)	1.05 (0.96 to 1.14)
VAS-pain remission					
3 months	No	1055 (79.0)	281 (21.0)	1.0 ref.	1.0 ref.
	Yes	710 (71.6)	282 (28.4)	1.25 (1.13 to 1.38)	1.13 (1.02 to 1.26)
6 months	No	892 (79.7)	227 (20.3)	1.0 ref.	1.0 ref.
	Yes	663 (75.4)	216 (24.6)	1.14 (1.02 to 1.28)	1.08 (0.96 to 1.22)
1 year	No	1115 (79.0)	297 (21.0)	1.0 ref.	1.0 ref.
	Yes	890 (73.6)	319 (26.4)	1.17 (1.07 to 1.28)	1.10 (1.00 to 1.21)
HAQ decrease over the median					
3 months	No	796 (76.9)	239 (23.1)	1.0 ref.	1.0 ref.
	Yes	842 (74.8)	283 (25.2)	1.05 (0.96 to 1.16)	1.11 (1.02 to 1.21)
6 months	No	706 (79.0)	188 (21.0)	1.0 ref.	1.0 ref.
	Yes	734 (76.6)	224 (23.4)	1.07 (0.96 to 1.18)	1.10 (0.99 to 1.21)
1 year	No	872 (78.6)	237 (21.4)	1.0 ref.	1.0 ref.
	Yes	949 (74.6)	323 (25.4)	1.11 (1.02 to 1.20)	1.15 (1.06 to 1.25)

Risk ratio crude: poisson regression estimates without adjustment. Risk ratio*: poisson regression estimates adjusted for age at the diagnosis, gender, alcohol assumption, pack-years of smoking, body mass index, and baseline outcome values. *DAS28* disease activity in 29 joints, *VAS* visual analog scale, *HAQ* health assessment questionnaire

### Risk ratios for outcome measures decrease over the median

Higher education was not associated with increased chance of improvement (more than the median) in VAS-pain (Table 4). Thus, patients with a university education had less pain at baseline and had equivalent decrease to patients without, leading to better pain outcomes at follow up. For HAQ, patients with a university education had a greater chance of improvement in physical function at the 1-year visit (1.11(1.02–1.20)); after adjustment the result was also significant at the 3-month visit (1.11 (1.02–1.21)).

In addition to all the analyses above, we performed analysis with extra adjustments for residential area, leisure time physical activity, ACPA-status, treatment at baseline and study phase, but the results did not alter substantially and those variables were not kept in the final analyses. We subsequently carried out the stratified analysis based on ACPA status, but found no differences between the two subgroups. We also performed stratified analysis based on treatments, and again found no differences.

## Discussion

In this population-based cohort with early RA, receiving standard care in a country with a tax-financed, generally accessible healthcare system, educational background affect neither the time to diagnosis nor treatment during the first year. Furthermore, we found that educational background was not associated with disease activity (DAS28), but patients with a high educational level had slightly less pain (VAS-pain) and less functional disability (HAQ) at baseline and during the first year receiving standard care. Consequently, they were also more likely to achieve pain remission and improvements in functional impairment.

Methodological advantages of our study are its large sample size, the population-based design, the inclusion of incident cases, and the extensive information available. These features made it possible to adjust for several potential confounders. The general welfare system in Sweden provides health care to all citizens irrespective of SES, and the similar symptom duration observed among patients with different educational levels indicates that the system does not systematically favor earlier attention to individuals with higher education. Limitations in this study include self-reported educational background, which could have introduced non-differential misclassification. This misclassification was probably small as participants can be expected to remember their highest achieved education relatively easily. Not all EIRA study patients were followed in the clinical quality register, SRQ. However, the proportion of university-educated patients in the EIRA study was very similar (23 % and 20 %) regardless of inclusion in the SRQ.

To some extent our results confirm previous findings on the relationship between low SES and worse disease outcome for some subjective measures [3, 6–11]. However, unlike previous reports, where fewer outcome measures were examined, we carried out a comprehensive investigation with both subjective (VAS-pain, VAS-global, tender joint count, HAQ) and objective outcomes (swollen joint count and the inflammatory marker, ESR). We were also able to record prescription of different anti-rheumatic therapies, and more importantly, adjusted for several potential confounders. Interestingly, we found that levels of education affected neither time to diagnosis nor prescribed anti-rheumatic treatments, and that patients with a low educational level were not inferior in terms of good disease control, but rather the perceived pain control and functional capacity. These results are consistent with the long-term results of the BARFOT study in which 2,800 patients with early RA were followed up in southern Sweden, with no significant effects found between socioeconomic status and treatment or outcomes (DAS28 and EULAR response) [16]. In this context it is important to note that the HAQ questionnaire is a patient-reported outcome (PROM), thus, it relies exclusively on how the patient perceives functional impairment [17]. The same applies for the VAS-pain scale, and it’s possible that educational background influences how one interprets such measures. Another possible explanation for the differing results for the subjective outcomes, or PROMs, could be unmeasured confounding, e.g., those with a low educational level might have a more strenuous workload. Given similar disease activity, patients with physically demanding jobs may experience and report more pain and disability in relation to their daily activities. Moreover, although patients with a high educational level had significantly lower VAS-pain at baseline than patients with a low educational level, from a statistical point of view, it should be noted that the difference between 49 and 53 on a 100-mm VAS is probably of limited clinical relevance. For the HAQ, the median values were 0.88 and 1.00, respectively, and it has previously been concluded that a difference >0.2 is needed for clinical relevance [18].

Despite accumulating evidence demonstrating the contrasting etiology between the two serologically defined RA groups based on ACPA [19], our findings showed no apparent difference in relation to ACPA-status. Several studies have reported low rates of DMARDs use in RA patients in general (30–52 %) [20–24]. One study consisting of 93,143 patients found that individuals with low income or SES received fewer DMARDs prescriptions [25]. Our findings in a country with evenly accessible healthcare, however, did not support that educational background affects the prescription of treatments, with similarly high rates of treatment with DMARDs, NSAIDs, corticosteroids, and biologic agents both at baseline and during the first year, among patients with distinct educational backgrounds.

## Conclusions

To summarize, we found that higher-educated newly diagnosed RA patients had less pain and less functional impairment at diagnosis and throughout the follow-up period, although those statistically significant differences were of limited clinical relevance. Consequently, the patients with a higher level of education had a slightly greater chance of achieving pain remission, and improvement in physical function. Our study demonstrates that RA patients with different educational levels have the same opportunity to access healthcare including treatment; and that in this context, educational background has a very limited influence on the disease course.

## Abbreviations

ACPA: anti-citrullinated protein/peptide antibodies
BMI: body mass index
DAS28: the 28-joint disease activity score
DMARD: disease-modifying anti-rheumatic drug
EIRA: Epidemiological investigation of rheumatoid arthritis
EULAR: European League Against Rheumatism
ESR: erythrocyte sedimentation rate
HAQ: health assessment questionnaire
NSAID: non-steroidal anti-inflammatory drug
PROM: patient-reported outcome measures
RA: rheumatoid arthritis
RR: risk ratio
SES: socioeconomic status
SRQ: Swedish Rheumatology Quality Register
VAS: visual analog scale
